# Effective Population Size, Extended Linkage Disequilibrium and Signatures of Selection in the Rare Dog Breed Lundehund

**DOI:** 10.1371/journal.pone.0122680

**Published:** 2015-04-10

**Authors:** Sophia Pfahler, Ottmar Distl

**Affiliations:** Institute for Animal Breeding and Genetics, University of Veterinary Medicine Hannover, Bünteweg 17p, 30559 Hannover, Germany; Oregon State University, UNITED STATES

## Abstract

The Lundehund is an old dog breed with remarkable anatomical features including polydactyly in all four limbs and extraordinary flexibility of the spine. We genotyped 28 Lundehund using the canine Illumina high density beadchip to estimate the effective population size (N_e_) and inbreeding coefficients as well as to identify potential regions of positive selection. The decay of linkage disequilibrium was slow with r^2^ = 0.95 in 50 kb distance. The last 7-200 generations ago, Ne was at 10-13. An increase of N_e_ was noted in the very recent generations with a peak value of 19 for N_e_ at generation 4. The FROH estimated for 50-, 65- and 358-SNP windows were 0.87, 087 and 0.81, respectively. The most likely estimates for F_ROH_ after removing identical-by-state segments due to linkage disequilibria were at 0.80-0.81. The extreme loss of heterozygosity has been accumulated through continued inbreeding over 200 generations within a probably closed population with a small effective population size. The mean inbreeding coefficient based on pedigree data for the last 11 generations (F_Ped_ = 0.10) was strongly biased downwards due to the unknown coancestry of the founders in this pedigree data. The long-range haplotype test identified regions with genes involved in processes of immunity, olfaction, woundhealing and neuronal development as potential targets of selection. The genes *QSOX2*, *BMPR1B* and *PRRX2* as well as *MYOM1* are candidates for selection on the Lundehund characteristics small body size, increased number of digits per paw and extraordinary mobility, respectively.

## Introduction

Recently, an extraordinary low genetic variability could be demonstrated for the Lundehund using both single nucleotide polymorphisms (SNPs) and microsatellite markers [1–3]. Two bottlenecks reduced the population size of this dog breed to five closely related individuals worldwide [4]. A local population of Lundehund survived in Måstad on the isolated island of Værøy. From this population in 1960, five animals founded the actual population according to anecdotal reports. Around the year 1960, some Lundehund were crossbred with Norsk Buhund. Progeny of the backcrossing with a fifteen-sixteenth proportion of Lundehund blood were registered as purebred Lundehund (http://www.lundehund.com/the%20lundehund.htm). Presumably, intensive selection was necessary to retain the Lundehund phenotype. This includes a polydactyly with six or more toes per each limb and additional foot pads for sure-footedness, closable ears, an extraordinary mobility of the forelimbs and a height at withers of 32 to 38 cm (http://www.fci.be/en/nomenclature/NORWEGIAN-LUNDEHUND-265.html).

To preserve this rare Nordic dog breed with special phenotypic characteristics is a demanding task for breeding associations. Understanding the genetic basis of this dog breed would help to establish a breeding program conserving desirable characteristics and controlling hereditary defects.

The objectives of the present study were to genotype 28 Lundehund using the canine Illumina high density beadchip (Illumina, San Diego, CA, USA) to estimate the effective population size (N_e_) from data on linkage disequilibria (LD). We identified runs of homozygosity (ROH) as regions with a local loss of genetic variation. Inbreeding coefficients were calculated on the basis of pedigree data (F_Ped_) and genotype information (F_ROH_, F_IS_) to compare the results of these different methods for this population. We applied the long-range haplotype test to search for potential selective sweeps.

## Results

**Fig 1:**
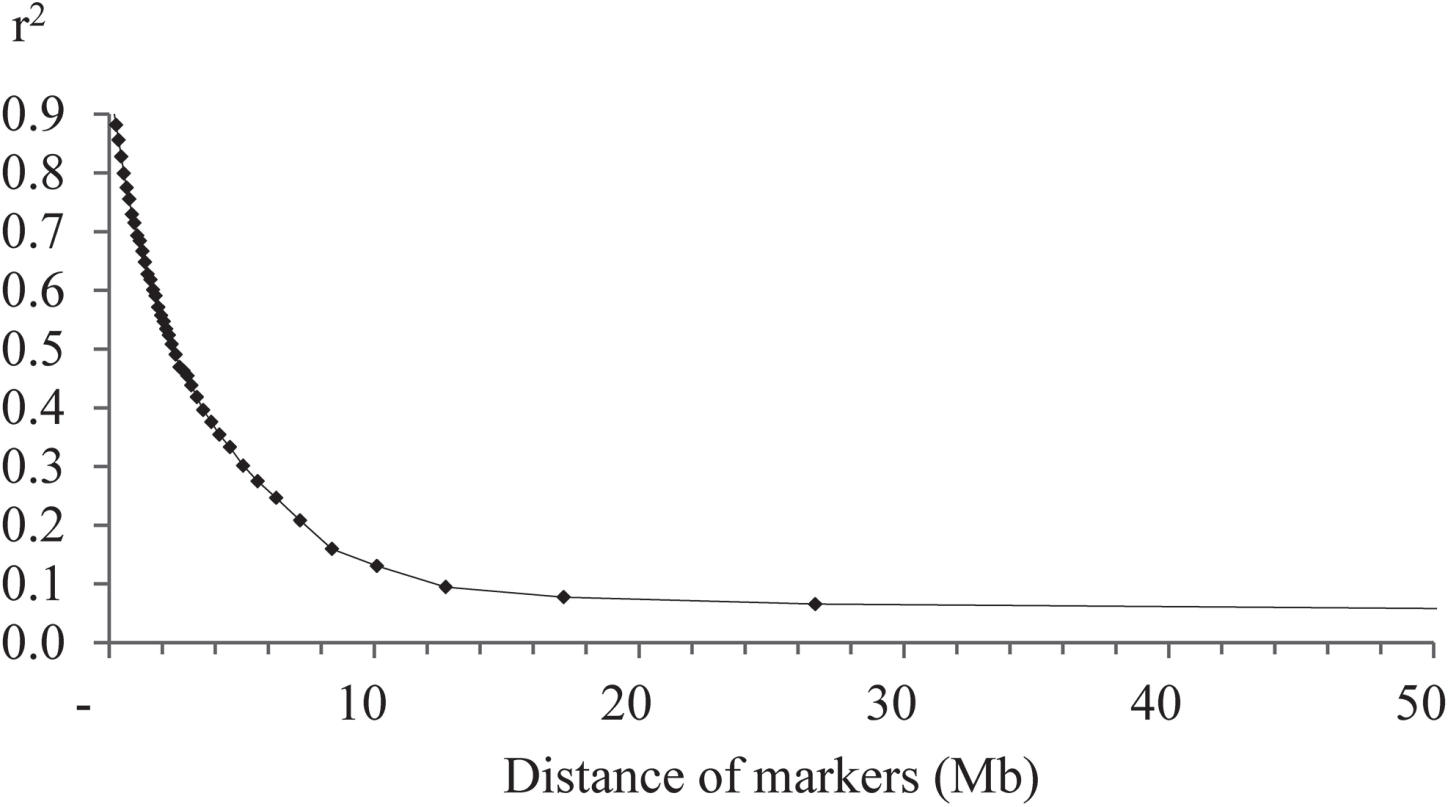
Decay of linkage disequilibria (r^2^) between SNP pairs spanning an increasing distance.

**Fig 2:**
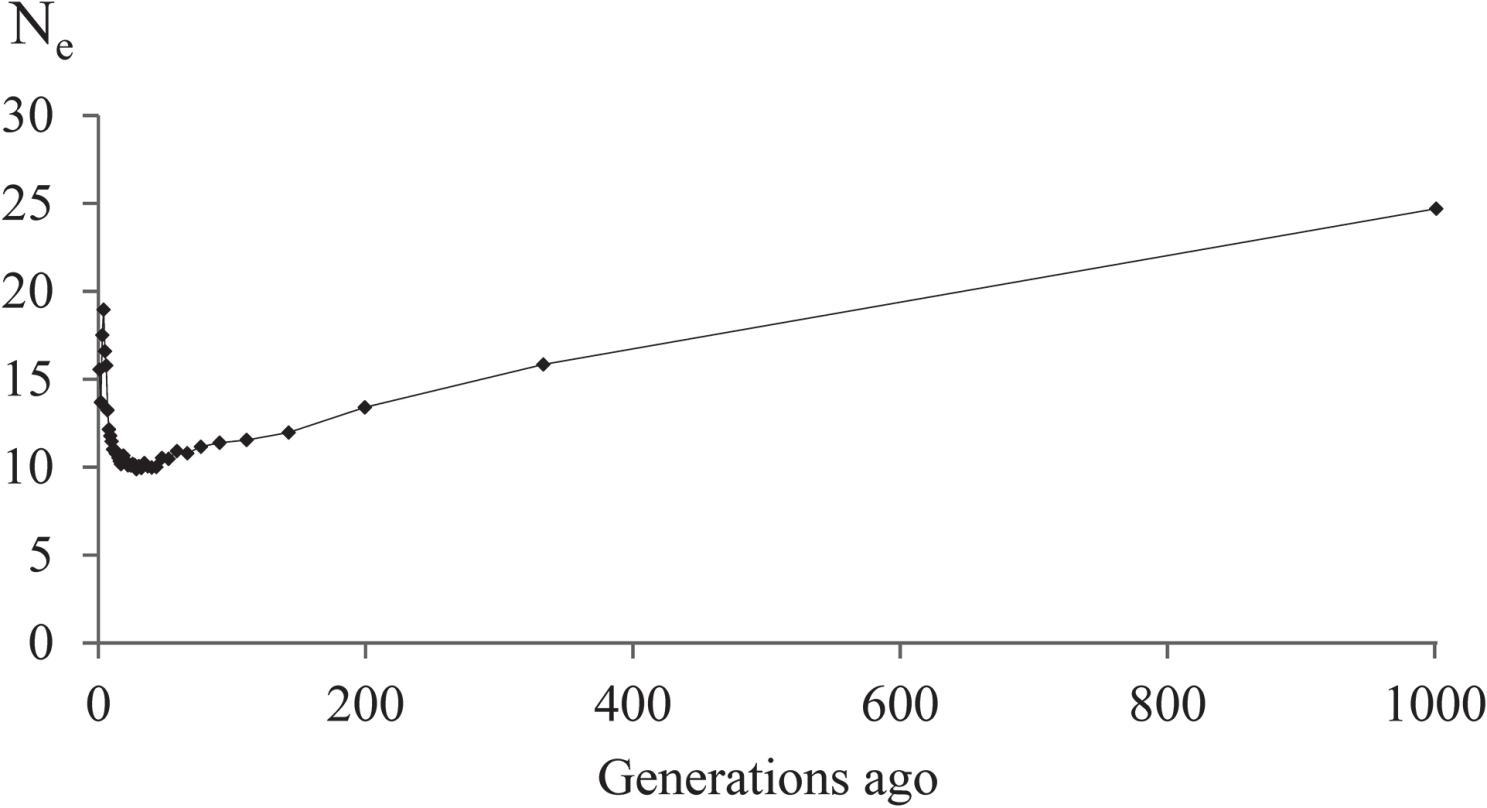
Ancestral population size of the Lundehund in the last 1000 generations. The effective population size (N_e_) was estimated from the mean r^2^ for the 38 canine autosomes.

**Fig 3:**
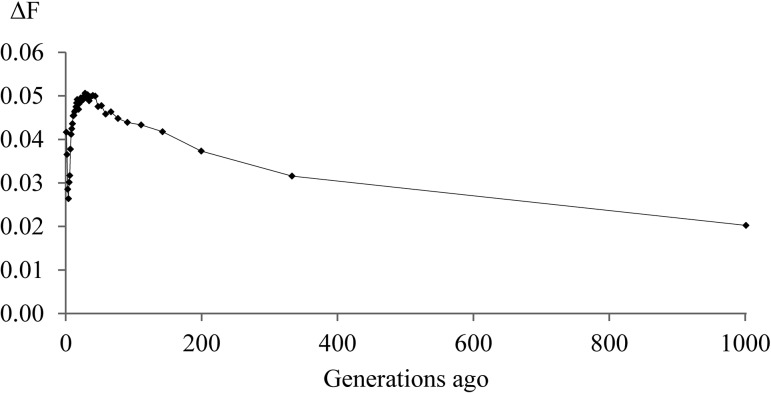
Average increase in inbreeding (ΔF) in the Lundehund for 1 to 1000 generations ago.

The mean r^2^ as a measure of LD was 0.95 and 0.91 in the Lundehund for SNPs 50.0 kb and 150 kb apart, respectively. The LD decreased to values below r^2^ = 0.25 for SNPs 6.3 Mb apart (Fig 1). The effective population size was estimated for the last 1000 generations corresponding to a marker distance of 0.05 to 50 Mb. The estimate for N_e_ 1000 generations ago decreased from 24.7 to 13.4 after 800 generations. Between 200 and 7 generations ago, N_e_ was on a constantly very slowly decreasing level at 10–13 (Fig 2). In the very recent generations, N_e_ recovered to a size of 19 four generations ago and fell again from this peak in generation four to N_e_ = 12 in generation 1 (S1 Fig). The increase in inbreeding per generation (ΔF) reached a maximum of 0.05 in the generations 7–200 ago. A slight decrease of ΔF to generation 4 ago and thereafter, again an increase in the most recent three generations could be noted (Fig 3 and S2 Fig).

**Fig 4:**
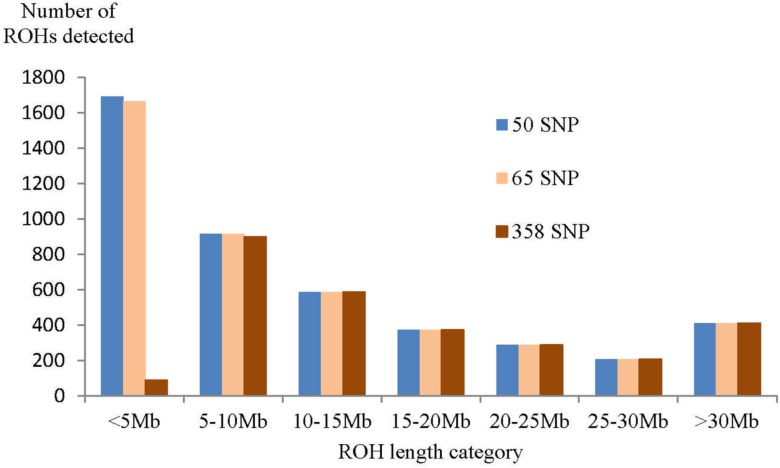
Number of runs of homozygosity (ROH) per length category for the different minimum SNP length thresholds used for ROH detection.

We expected a high level of inbreeding in this small population when calculating the inbreeding coefficients F_ROH_ based on ROH information and F_IS_ (fixation coefficient). The minimum length of a ROH in SNPs was set to 50 or 65 according to the recommendations of a simulation study in man with a high density SNP-array and approximately 0.65 of all 669,219 SNPs with a MAF >0.01 [5]. Using these thresholds, we identified 393 and 390 consensus ROHs common to all 28 Lundehund, respectively (S1 Table). The average cumulative length of all ROHs per individual was 1.99 Gb (Table 1), corresponding to 87% of the autosomal canine genome (2.29 Gb, CanFam3.1). However, one must keep in mind, that the probability to identify a ROH by chance due to high LD and non-IBD segments was higher in the Lundehund in comparison to man because of its extraordinary high homozygosity [3]. In order to reduce the type I error rate for the detection of ROHs due to the high homozygosity of SNPs in Lundehund, we introduced a threshold of 358 SNPs as the minimum number of consecutive homozygous SNPs in a ROH. This minimum number of SNPs that constituted a ROH was calculated for the present data set taking into account the mean homozygosity of SNPs, the total number of SNPs and individuals in the analysis and the percentage of false positive ROHs [6,7]. The 50-SNP-, 65-SNP- and 358-SNP-threshold resulted in ROHs of at least 500 kb, 780 kb and 4.3 Mb. With higher thresholds for SNPs constituting a ROH, the number of ROHs <5Mb that could be detected was markedly reduced and thus, their overall mean length was increased (Table 1). Accordingly the analysis of ROH length categories for the different thresholds revealed that there was a clear difference in the number of ROHs detected for the length category <5Mb, but only a small difference in the number of ROHs detected for the length category 5–10 Mb and no differences for the length categories >10 Mb (Fig 4). The shortest ROH identified in one animal had a length of 1.0 Mb for the 50-SNP- threshold and of 4.3 Mb for the 358-SNP-threshold. The mean F_ROH_358SNP_ for all Lundehund was 0.81 (0.53 to 0.86) and both, the F_ROH_50SNP_- and F_ROH_65SNP_-values, were 0.87 (0.83 to 0.90).

**Table 1:** Three different length thresholds were set for the calling of runs of homozygosity (ROH).

Thresholds	ROHs per individual				Consensus ROHs			
Minimum ROH length in SNP	Mean number of ROHs	Length of shortest ROH (Mb)	Mean length of ROH (Mb)	Length of all ROHs (Gb)	Number of consensus ROHs	Mean length of consensus ROH (Mb)	Length of all consensus ROHs (Gb)	Mean F_ROH_
50	163	1.0	13.3	1.99	393	3.4	1.35	0.87
65	162	1.0	13.4	1.99	390	3.5	1.35	0.87
358	103	4.3	18.3	1.84	108	7.0	0.76	0.81

For all thresholds the number and length of detected ROHs per individual and detected consensus ROHS are given, respectively. A consensus ROH was defined as the common proportion of ROHs that overlapped in all 28 Lundehund. The mean inbreeding coefficient F_ROH_ was estimated depending on the different thresholds.

Setting minimum ROH lengths for the 358-SNP-threshold to 1–10 Mb (representing the latest 50 to 5 generations), short ROHs due to LD and probably most non-IBD segments have been discarded. The values for F_ROH_358SNP>1–10 Mb_ showed correlations at 1.00–0.98 (P<0.001) with F_ROH_358SNP_ (S2 Table). The values for F_ROH_358SNP >1–10 Mb_ were at 0.70–0.81.

Similar results for F_ROH_50SNP_ and F_ROH_65SNP_ were obtained for the same restrictions to the size of ROHs. Correlations for both, F_ROH_50SNP>1–10 Mb_ and F_ROH_65SNP>1–10 Mb_ were at 0.64–0.65 (>10 Mb) to 1.0 (>1 Mb) and also the values for both, F_ROH_50SNP>1–10 Mb_ and F_ROH_65SNP>1–10 Mb_ were at 0.70 (>10 Mb) to 0.87 (>1 Mb).

The mean F_IS_-value was -0.07 and had a significant correlation of 0.88 (P<0.001) with both F_ROH_50SNP_ and F_ROH65_SNP_. The correlation of F_IS_ with F_ROH_358SNP_ was at 0.29 (P = 0.12) because all ROHs <358 SNPs were not regarded when calculating F_ROH_358SNP_. The mean pedigree inbreeding coefficient F_Ped_ calculated for the 28 Lundehund for the last 11 generations with completeness of pedigrees of 97.3% was 0.10 (ΔF = 0.01 per generation) and 0.01 for 5 generations with 100% completeness. We found a negative correlation of -0.47 between F_IS_ and F_Ped_ (P<0.05) and a slight positive correlation (0.11) between F_ROH_358SNP_ and F_Ped_.

The analysis for extended haplotype homozygosity (EHH) revealed 15 core regions on ten chromosomes with significant values for the relative extended haplotype homozygosity (REHH) and a haplotype frequency of at least 0.50 (Table 2). On canine chromosomes (CFA) 5, 7 and 9, pairs of core haplotypes are proximate to each other and therefore could possibly represent single selective sweep. In the genes located in the core regions, gene ontology terms linked to the thrombospondin, type 1 repeat and the lipocalin family were overrepresented with Bonferroni significance. We identified the genes Q*uiescin Q6 sulfhydryl oxidase 2* (*QSOX2*) at 49,213,295–49,245,232 bp and *LIM homeobox 3* (*LHX3*) at 49,248,643–49,254,118 bp on CFA9 as two potential candidates for selection on the small body size in the Lundehund (all coordinates for genes given on CanFam3.1). The genes *bone morphogenetic protein receptor*, *type IB* (*BMPR1B*) at 17,819,265–17,978,113 bp on CFA32 and *paired related homeobox* 2 (*PRRX2*) at 54,091,700–54,224,620 bp on CFA9 in two regions containing long-range homozygous haplotypes were previously reported as candidate genes for polydactyly in mice. A potential candidate gene for the selection on the extraordinary mobility of the Lundehund is *myomesin1* (*MYOM1*) at 69,701,355–69,834,023 bp on CFA7.

**Table 2:** Chromosomal regions with increased relative extended haplotype homozygosity (REHH) indicative for recent positive selection in the Lundehund.

CFA	Start base	End base	Haplotype	P	Number	Candidate	Associated function or phenotype	Previous	Position SNP
	(bp)	(bp)	frequency		of genes	gene	of candidate gene	study	(bp)
3	85,293,274	85,431,252	0.55	3.50	9	*SOD3*	Oxidative stress protection [8]	[9]	89,065,531
5	51,991,305	52,146,695	0.75	3.73	3	*DAB1*	Learning and memory [10]	[11]	55,658,719
5	52,178,854	52,220,901	0.75	3.88	3	*C8A*, *C8B*	Membrane attack complex [12]	[9]	57,774,351
7	70,189,846	70,282,222	0.78	3.29	5	*MYOM1*	Muscle elasticity [13]	-	-
7	70,358,563	70,427,583	0.78	3.29	4	*TGIF1*	Anterior neural differentiation in	-	-
							mice [14]		
9	49,289,547	49,353,060	0.83	15.18	41	*QSOX2*	Adult height [15]	-	-
9	49,399,638	49,465,271	0.83	4.04	41	*CAMSAP1*	Neurite outgrowth [16]	-	-
9	53,543,060	53,762,191	0.55	3.77	20	*NCS1*	Synaptic activity [17]	-	-
11	61,984,984	62,046,643	0.78	4.38	3	*TMEM38B*	Intracellular Ca2+ handling [18]	-	-
18	14,285,799	14,529,306	0.80	3.23	8	*SYPL1*	Transport vesicles [19]	[9,20]	16,591,125;
									18,141,067
25	46,108,914	46,136,878	0.70	5.38	4	-	-	-	-
25	49,556,362	49,731,926	0.70	3.01	15	*HDAC4*	Memory [21]	-	-
31	34,331,543	34,331,543	0.50	3.04	9	-	-	-	-
32	18,054,752	18,292,540	0.55	3.40	2	*UNC5C*	Tumor suppressor [22]	-	-
33	9,705,043	9,935,813	0.50	3.01	3	-	-	-	-

The canine autosome (CFA), the chromosomal positions in base pairs (bp) for the canine assembly CanFam3.1, the frequencies of the candidate selected core haplotype, the—log_10_P-values (P) for the REHH and number of genes annotated within the core haplotype region are given. The nearest potentially selected gene and its associated function or phenotype in man or mice is indicated. The regions identified in the present study were compared to the results of previous studies regarding positive selection in dogs. Indicated is the position (CanFam2.0) of the SNPs previously supposed to be selected.

## Discussion

The Lundehund featured a clearly higher mean LD (r^2^ = 0.95) for SNPs in 50 kb distance in comparison to previous reports [23,24]. The distance at which r^2^ fell below 0.25 in the Lundehund (6.3 Mb) was longer in comparison to the Golden Retriever (200–500 kb), the Rottweiler (500–1000 kb) and the Newfoundland dog (100–200 kb) [23]. The low N_e_ for the last 7–200 generations indicated that the Lundehund already was a genetically small and closed population at a very early stage. This seems to be likely since the dogs were bred for a long time as valuable working dogs and were restricted to the coast area of northern Norway because of this task. The most likely explanation for the increasing N_e_ and the decreasing ΔF in the recent 4–6 generations was crossbreeding as reported by Lundehund breeders (http://www.lundehund.com/the%20lundehund.htm). However, the use of a few crossbred Lundehund did not increase N_e_ for more than 2–3 generations and accordingly a substantial and longer lasting improvement of the genetic variability was not achieved for the Lundehund. Particularly, repeated backcrossings and selection for the Lundehund phenotype might have quickly eroded the newly introduced genetic variability.

The inbreeding coefficients based on 11 generations of pedigree information were biased downwards because founders seemed strongly related. Restricting the pedigree information to 5 generations considerably decreased the inbreeding load registered by the estimated F_Ped_-values. The F_Ped_ is a measure of IBD status for the individuals of a pedigree assuming unrelated founders. There were low correlations between F_Ped_ and F_ROH_ as well as F_IS_ indicating large variation of F_ROH_ and F_IS_ for individuals with the same F_Ped_. We assume strong relationships among the founders of the pedigree data due to the small N_e_ over 200 generations. Similar observations were made for F_Ped_ and F_ROH_ in cattle [25,26]. However, F_Ped_ and F_ROH_ did not diverge in cattle at such an extent like in Lundehund and F_ROH_ in cattle was much lower than in the Lundehund investigated here [25–27]. The most likely explanation is the extreme homozygosity in the Lundehund genotyped here due to breeding closely related dogs over a very long time. Therefore, the pedigree information of the registration certificates cannot be recommended for mating decisions most of the time.

Of the inbreeding coefficients based on SNP data, the F_ROH_ was reported as the most effective method to distinguish between IBD and IBS [28]. A high minimum SNP threshold for the calling of a ROH could reduce the probability to detect regions homozygous by chance [6], but reduces the number of generations considered for the estimation of inbreeding [28]. We used the moderate thresholds 50 and 65 SNPs and an extreme threshold of 358 SNP to take into account the low heterozygosity in the Lundehund. The 50-SNP-, 65-SNP- and 358-SNP-threshold resulted in ROHs of at least 500 kb, 780 kb and 4.3 Mb. This meant that inbreeding in the previous 100, 64 and 12 generations was captured or the base populations used for determining F_ROH_ were 100, 64 and 12 generations back. This considerable restriction in generations considered for calculating F_ROH_ still resulted in a mean F_ROH_358SNP_ similar (ΔF_ROH_ = 0.06) to the mean F_ROH_50SNP_ and F_ROH_65SNP_. Nevertheless, the correlation between F_ROH_ and F_IS_ for the 50 and 65 SNPs thresholds decreased from 0.88 to 0.29 for the 358-SNP-threshold. This demonstrates that the 358-SNP-threshold may be less influenced by chance or single homozygous SNPs. We therefore assumed that the extreme level of homozygosity in the Lundehund with 87% of the genome covered by ROHs made it impossible to completely differentiate between IBS- and IBD-segments. The level of inbreeding might be slightly overestimated using F_ROH_50SNP_ and F_ROH_65SNP_ thresholds. Restricting minimum length of ROH to >10 Mb, gave still estimates for F_ROH_ at 0.70, independent of the SNP-threshold. The genetic variability of more polymorphic microsatellite markers was also found to be consistently low in the Lundehund in previous studies [2,3]. However, we cannot preclude some ascertainment bias of the beadchip. The F_ROH_ values of the individuals were suggested as a measure of inbreeding in conservation programs [29]. Our results indicated that the distinction between IBD and IBS could be difficult if there is a very low variability present in the respective population and the length of ROHs used for estimating genomic inbreeding coefficients may be critical. In case of the Lundehund, the very small N_e_ over 200 generations due to geographical isolation and historical bottleneck inevitably led to more consanguineous matings and to an increase of the lengths of IBD-segments through common ancestors with increasing inbreeding. This might be the reason why restrictions of ROHs from >1 Mb up to >10 Mb (representing 5 generations back) gave very similar estimates for genomic inbreeding coefficients.

The long and frequent ROHs made it difficult to identify signatures of selection by the reduction of local genetic variability, since most consensus ROHs had possibly arisen due to bottleneck or inbreeding effects and not due to selective pressure. This assumption was confirmed by the results of a study detecting ROHs in pigs [30]. We therefore chose the long-range haplotype test reported as robust to ascertainment bias [31] to identify regions that are potentially positively selected in the Lundehund. The important limitation of this test is that haplotypes with an allele frequency <0.1 are potentially missed [31]. This particularly applies to the haplotypes underlying breed defining traits that are completely fixed in pure bred dogs.

The potential selected regions on CFA3, 5 and 18 overlapped with candidate selected regions identified by previous studies [9,11,20]. Two terms were enriched in the functional annotation clustering analysis for the regions with long-range haplotypes. These terms were associated with wound healing and neuronal development [32,33] (thrombospondins) and olfaction and immune response [34] (lipocalins). The categories “olfaction”, “wound healing” and “immunity” were known targets of selection; they were enriched in human selective sweeps [35,36] and “immunity” and “neuron differentiation” in candidate selected genes of the dog [11,37]. The olfactory sense and wound healing seemed to be logical targets of selection in hunting dogs that were bred for performance, high resilience and easy maintenance.

As a consequence of the long-range haplotype test in the present study, these haplotypes occurred in a high frequency, but were not fixed in the Lundehund. Genes contained in this region may still be under selection for Lundehund characteristics up to date. Assuming previous crossbreeding, this is especially true for genes encoding breed defining traits that have lost their original fixation. We identified one candidate gene 44 kb from the core haplotype on CFA9 at 49.3 Mb with the highest—log_10_P-value. *QSOX2* on CFA9 at 49.2 Mb is the canine homologue of a human gene associated with adult height [15]. According to our knowledge this is the first time *QSOX2* has been linked to canine body size. Another candidate gene influencing the height at withers is *LHX3* on CFA9 at 49.2 Mb. In German shepherd dogs, a 7-bp deletion of this gene causes a combined pituitary hormone deficiency including pituitary dwarfism [38]. Other mutations in *LHX3* might lead to a reduction of height without associated health problems. Body size is a selection objective in nearly all breeding standards. It seems to be especially important and thus a potential target of selection in the Lundehund, which is bred for moving in spatially limited breeding cavities of puffins [4]. The gene *spermatogenesis and oogenesis specific basic helix-loop-helix 1* (*SOHLH1* on CFA9 at 49.6 Mb) near the second core region encodes a transcription factor essential for spermatogenesis that is a known target of selection [35,36,39]. Interestingly, the region at 49.113–49.150 Mb on CFA9 is homologue to a human genomic region that is associated with inflammatory bowel disease and contains the candidate gene *caspase recruitment domain family*, *member 9* [40]. A high frequency of protein-losing gastroenteropathies is reported for the Lundehund [41]. Selection for body size or male fertility might have increased the frequency of haplotypes disadvantageous for the intestinal balance and thereby disposed the whole breed to gastroenteropathies. Two candidate selected genes for the polydactyly characteristic in the Lundehund were found near the long-range haplotypes. Defects of the gene *BMPR1B* on CFA32 at 17.9 Mb led to a praeaxial polydactyly in mice [42]. Double loss of function mice for both *paired related homeobox* genes *PRRX1* and *PRXX2* (*PRRX2* on CFA9 at 54.1–54.2 Mb) also displayed postaxial polydactyly [43]. The genes *aristaless-like 4* and *limb development membrane protein 1* known for polydactyly in dogs [44,45] were not included in the candidate selected regions identified in the Lundehund. A potential candidate gene for selection on the extraordinary mobility of the Lundehund is *MYOM1* on CFA7 that acts as an elastic ribbon in the central M-band of muscle sarcomeres [13].

### Conclusions

The Lundehund very likely descended from a small founder population with an effective population size <20 for the last 200 generations. The high F_ROH_ reflected the extreme loss of genetic variability in this breed. We identified 15 regions with long-range haplotypes that were potentially under positive selection in the Lundehund. New potential candidate genes for polydactyly and body size in dogs including *PRRX2*, *BMPR1B* and *QSOX2* were identified. Selection for body size or male fertility might have predisposed the Lundehund to gastroenteropathies.

## Materials and Methods

### Ethics statement

Animal welfare committee approval was not obtained according to the German Animal Welfare Law (released on 05/18/2006, last changes on 07/08/2013) because all data was extracted from an existing database and EDTA-blood samples from the bio-bank of a diagnostic lab. The blood samples were collected and sent in by accredited veterinarians according to the national and international guidelines for animal welfare.

### Samples and genotyping

Blood samples of 28 Lundehund were taken from the bio-bank of the Institute for Animal Breeding and Genetics at the University for Veterinary Medicine Hannover. Twelve of the dogs had been used for a previous analysis of genetic variability in this breed [3]. The pedigree data for all dogs was provided by their owners. We extracted genomic DNA from the blood samples through a standard ethanol fractionation with concentrated sodiumchloride (6 M NaCl) and sodium dodecyl sulphate (10% SDS). The concentration of DNA was adjusted to 50 ng/μl per sample. Genotyping was done on the canine Illumina high density beadchip containing 173,662 SNPs.

### NCBI GEO submission

Raw and processed data is available for the 28 Lundehund with genotypes and can be obtained through the GEO website (http://www.ncbi.nlm.nih.gov/geo/) using accession number GSE66677.

### Statistical analysis

The dataset for the estimation of r^2^-values and N_e_ consisted of 162,557 SNPs with a genotyping rate >0.90 in all 28 Lundehund. For the detection of ROHs we excluded all SNPs from sex chromosomes resulting in a reduced dataset of 157,423 autosomal SNPs. A summary on the diversity of SNPs in the Lundehund genotyped is given in S3 Table. For the long-range haplotype test this data was additionally phased and missing genotypes were imputed using BEAGLE 3.3.2 [46]. We did not remove SNPs through MAF and light-to-moderate pruning prior to analysis because SNP coverage was uniform on the canine Illumina high density beadchip. In addition, we assumed all SNPs genotyped with a call rate >0.90 represent canine variants present in large across-breed panels. We checked this assumption and applied a filtering for MAF based on a multibreed panel as proposed in a few previous analyses [25–27]. The multibreed panel has been described previously and briefly, contained 1092 dogs of 14 breeds other than Lundehund with at least 12 animals per breed [3]. Removing SNPs due to a MAF<0.01 and <0.05 in the present data set for Lundehund resulted in 143,940 and 131,612 SNPs, respectively. The effects on r^2^, N_e_ and F_ROH_ due to the SNP-selection according to a MAF were very small and the estimates of the parameters after using a MAF-restriction mostly indistinguishable from the estimates without considering a MAF (S3–S6 Figs). The 50-SNP-, 65-SNP- and 358-SNP-threshold and a MAF-restriction <0.01 gave estimates at 0.87, 0.87 and 0.79 for F_ROH._ The corresponding values for F_ROH_ and a MAF-restriction <0.05 were 0.87, 0.87 and 0.78.

We calculated r^2^ as a measure of LD among SNP alleles per chromosome using PLINK (http://pngu.mgh.harvard.edu/purcell/plink) [47]. The r^2^–values for SNP pairs with 1 kb to 33.3 Mb distance between each other were grouped into distance bins of 0.1 Mb. For each bin the mean r^2^-values were calculated and the effective population size was estimated as *N_e_* = (1- *r^2^*)/(*4cr^2^*) with c = recombination rate in Morgan units [48]. Regarding the distance c between two SNPs we assumed that 100 Mb = 1 Morgan. The number of generations in the past was estimated as and rounded to the nearest integer. The expected length of IBD-DNA segments follows an exponential distribution with mean equal to 1/(2g), with g = the number of generations since the common ancestor [49]. The increase in inbreeding was computed as ΔF = 1/(2N_e_) [49].

The sliding windows for ROH detection with the program PLINK contained 50 or 65 SNPs. A ROH in one individual was called if a homozygous stretch contained 50 or 65 or more SNPs and extended over 1000 kb [5,7]. We did not allow for heterozygous SNPs and only for five missing SNP genotypes per homozygous region [5]. The matching proportions of ROHs overlapping in all Lundehund were pooled to consensus ROHs. The number of SNPs in the homozygous stretch was increased to 358 in a third analysis to reduce the type I error [6,7]. This threshold (l) was calculated as *l* = log*( α /(n_SPN_ x n_i_))* / log(hom), with an error rate ɑ of 0.05, a mean number of SNPs per individual n_SNP_ = 168,467, a mean homozygosity (hom) = 0.95 and the number of individuals n_i_ = 28 [6,7]. Mean homozygosity using 168,467 SNPs was calculated from the data set of all Lundehund.

The inbreeding coefficient F_ROH_ for each dog was estimated as the length of all ROHs in the respective individual divided by the total length of all autosomes covered by SNPs [50]. We used PLINK to calculate F_IS_-values for each individual i as F_IS,i_ = (O_i_-E_i_)/(n_SNP,i_-E_i_), with E_i_ = number of expected homozygous SNPs, O_i_ = number of observed homozygous SNPs and n_SNP,i_ = number of all SNPs genotyped in the respective individual [46]. The pedigree inbreeding coefficients (F_Ped_) for the 28 Lundehund were estimated using OPTI-MATE, version 3.88 (http://www.tiho-hannover.de/?id=3442) [51] considering the last 5 and 11 generations of pedigree information

Since it was difficult to differentiate if the consensus ROHs had their reduced genetic variability due to positive selection or inbreeding following the bottleneck, we used another method to identify potential selective sweeps in the Lundehund. The long-range haplotype test detects haplotypes that are long and frequent because they have risen to high frequency without time for recombination [31]. We calculated EHH values for core haplotypes using the program SWEEP [52]. The EHH values were corrected for the variability in recombination rate using the REHH statistic. The REHH values were grouped in 20 bins depending on the frequency of their corresponding core haplotypes. For each bin, P-values were obtained by log-transforming the EHH and REHH to achieve normality and calculating the mean and standard deviation [52]. The top five percent of haplotypes had—log_10_P-values >3 and were considered to be significant.

### Annotation analysis

The Database for Annotation, Visualization and Integrated Discovery (DAVID 6.7) (http://david.abcc.ncifcrf.gov/home.jsp) [53] was used for a first functional annotation analysis. We extended the core haplotypes with significant REHH by 0.5 Mb in both directions. All genes overlapping these regions in the canine assembly BROAD2 of Ensembl (http://may2012.archive.ensembl.org/Canis_familiaris/Info/Index) were considered for the analysis. Annotation terms represented in the functional annotation chart for the canine genes were tested for enrichment in a modified Fisher Exact test provided by DAVID. We took a closer look at the canine genes of the regions with long-range haplotypes using the databases Gene and OMIM (http://www.ncbi.nlm.nih.gov). All identified regions were checked against with the locations of canine homologues of genes known to be associated with polydactyly in humans [54].

## Supporting Information

S1 Fig Ancestral population size of the Lundehund in the last 50 generations. The effective population size (N_e_) was estimated from the mean r^2^ for the 38 canine autosomes.
(TIF)

S2 Fig Average increase in inbreeding (ΔF) in the Lundehund for 1 to 50 generations ago. (TIF)

S3 Fig Decay of linkage disequilibria (r^2^) between SNP pairs spanning an increasing distance after filtering for a minor allele frequency (MAF) <0.01 in a multibreed dog panel. The black graph shows the decay of linkage disequilibria without restriction due to MAF and the dark yellow graph represents the decay of linkage disequilibria after restriction due to a MAF<0.01.
(TIF)

S4 Fig Decay of linkage disequilibria (r^2^) between SNP pairs spanning an increasing distance after filtering for a minor allele frequency (MAF) <0.05 in a multibreed dog panel. The black graph shows the decay of linkage disequilibria without restriction due to MAF and the dark yellow graph represents the decay of linkage disequilibria after restriction due to a MAF<0.05.
(TIF)

S5 Fig Ancestral population size of the Lundehund in the last 1000 generations. The effective population size (N_e_) was estimated from the mean r^2^ for the 38 canine autosomes and after filtering for a minor allele frequency (MAF) <0.01 in a multibreed dog panel. The black graph shows N_e_ without restriction due to MAF and the red graph represents N_e_ after restriction due to a MAF<0.01.
(TIF)

S6 Fig Ancestral population size of the Lundehund in the last 1000 generations. The effective population size (N_e_) was estimated from the mean r^2^ for the 38 canine autosomes and after filtering for a minor allele frequency (MAF) <0.05 in a multibreed dog panel. The black graph shows N_e_ without restriction due to MAF and the red graph represents N_e_ after restriction due to a MAF<0.05.
(TIF)

S1 Table The proportions of the runs of homozygosity (ROHs) (threshold for ROH detection: >49 SNPs) that overlapped in all 28 Lundehund were pooled to consensus ROHs. For each consensus ROH the canine chromosome, the position of the start and end SNP in CanFam2.0, the number of SNPs included and the genes included to the region according to the Gene database (http://www.ncbi.nlm.nih.gov/gene/) is given. Note that no minimum threshold was set for the length of a consensus ROH (Exel data sheet).
(XLSX)

S2 Table Runs of homozygosity (ROHs) for 50-, 60- and 358-SNP-thresholds and with different restrictions for minimum lengths of ROHs in Mb (MIN-ROH). Given are the minimum length of ROHs, their equivalence in number of generations back (GEN), the inbreeding coefficients F_ROH_50SNP,_ F_ROH_65SNP and_ F_ROH_358SNP_ with their standard deviations (SD) and Pearson correlation coefficients with the respective F_ROH_ without restrictions for minimum length.
(DOCX)

S3 Table Genetic diversity of Lundehund genotyped using the Illumina Canine High Density Beadchip. The mean minor allele frequency (MAF) over all 157,423 autosomal SNPs in the Lundehund, the proportion and mean number of informative SNPs (MAF >0.0), the fixation index (F_IS_) and the mean number of observed and expected homozygous SNPs are given.
(DOCX)
