# Supplementary figures and images for: Effective Population Size, Extended Linkage Disequilibrium and Signatures of Selection in the Rare Dog Breed Lundehund
Source: PLoS One. 2015 Apr 10;10(4):e0122680. doi: 10.1371/journal.pone.0122680 (PMC4393028; doi:10.1371/journal.pone.0122680)

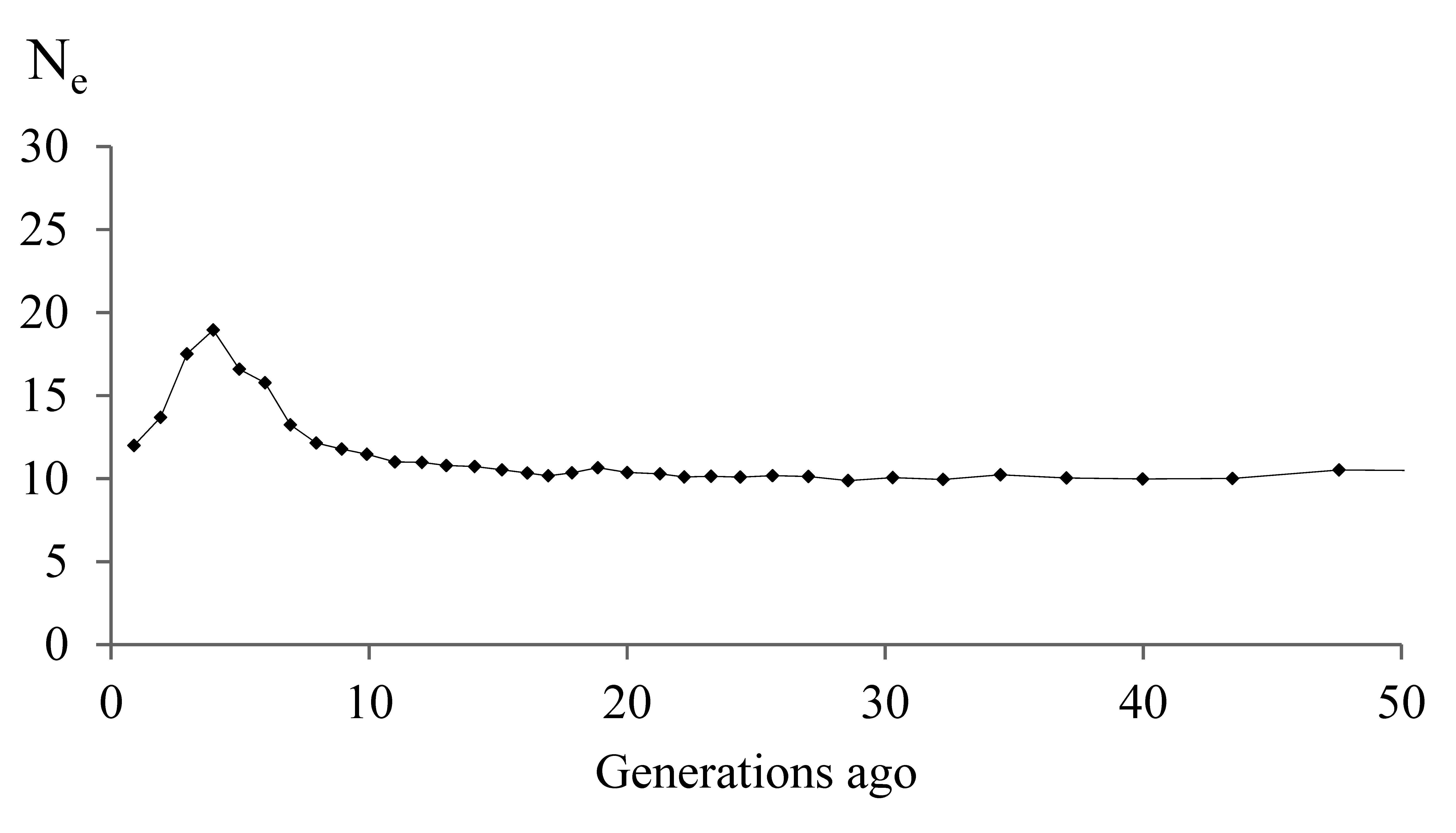

Supplement: S1 Fig — The effective population size (Ne) was estimated from the mean r2 for the 38 canine autosomes. (TIF) [file pone.0122680.s001.tif]

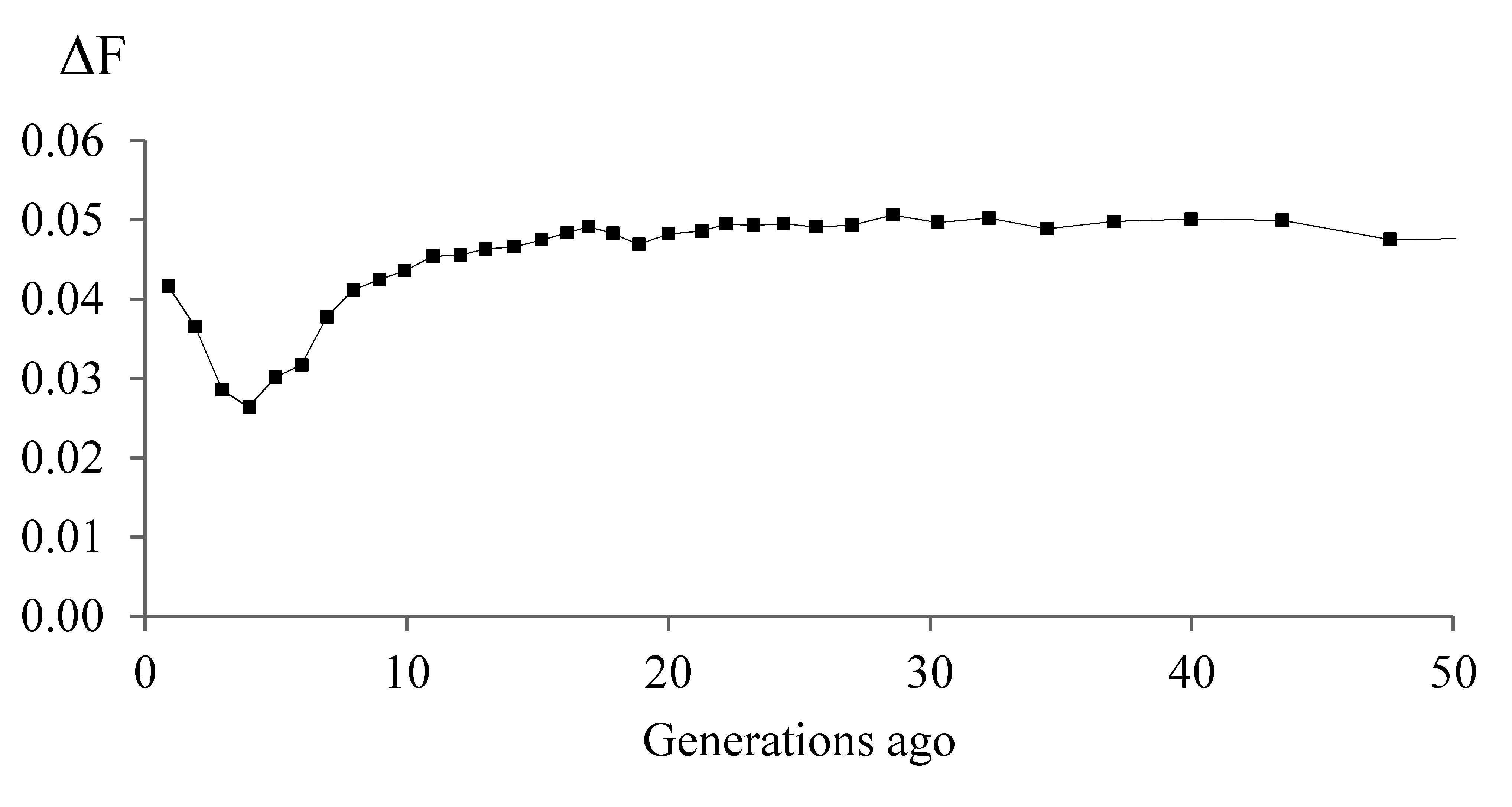

Supplement: S2 Fig — (TIF) [file pone.0122680.s002.tif]

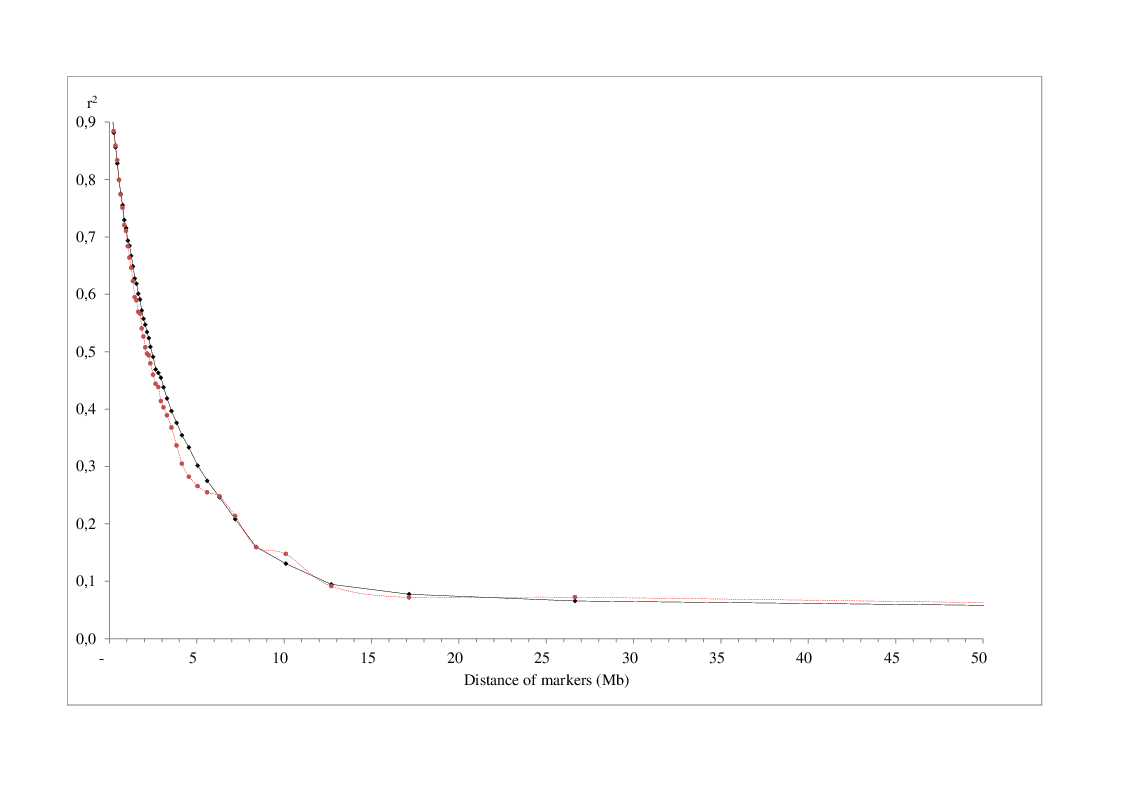

Supplement: S3 Fig — The black graph shows the decay of linkage disequilibria without restriction due to MAF and the dark yellow graph represents the decay of linkage disequilibria after restriction due to a MAF<0.01. (TIF) [file pone.0122680.s003.tif]

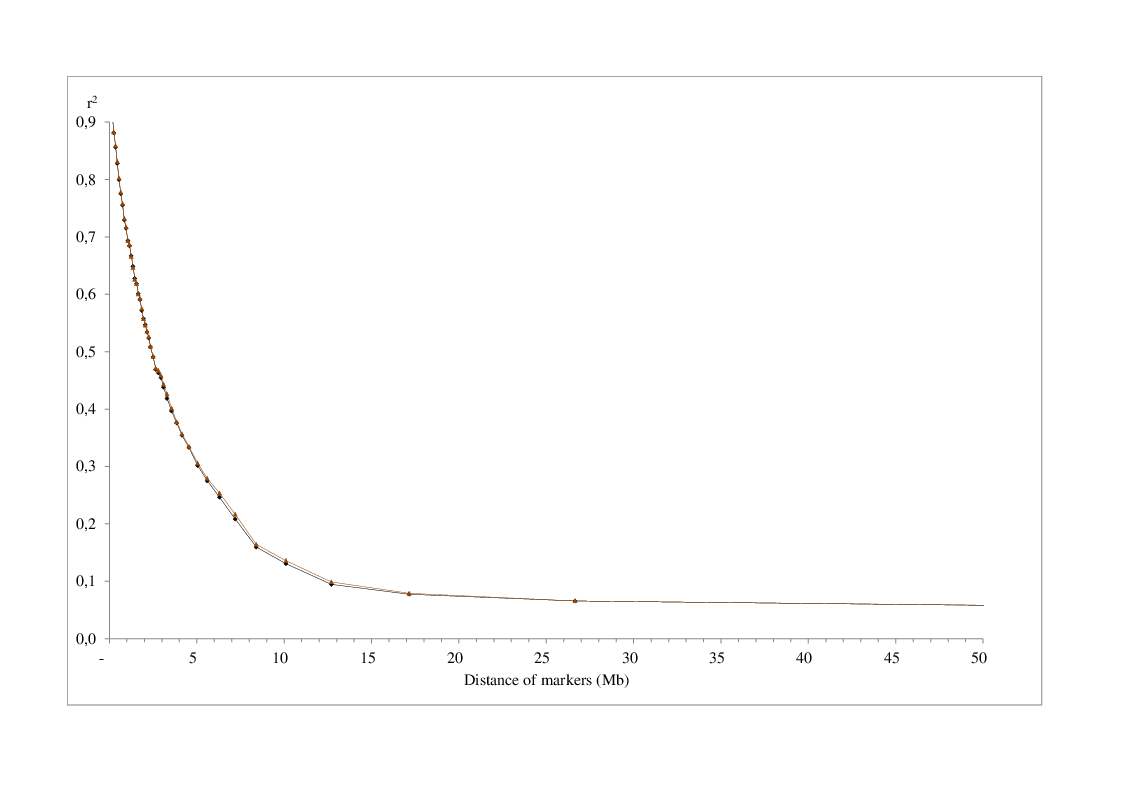

Supplement: S4 Fig — The black graph shows the decay of linkage disequilibria without restriction due to MAF and the dark yellow graph represents the decay of linkage disequilibria after restriction due to a MAF<0.05. (TIF) [file pone.0122680.s004.tif]

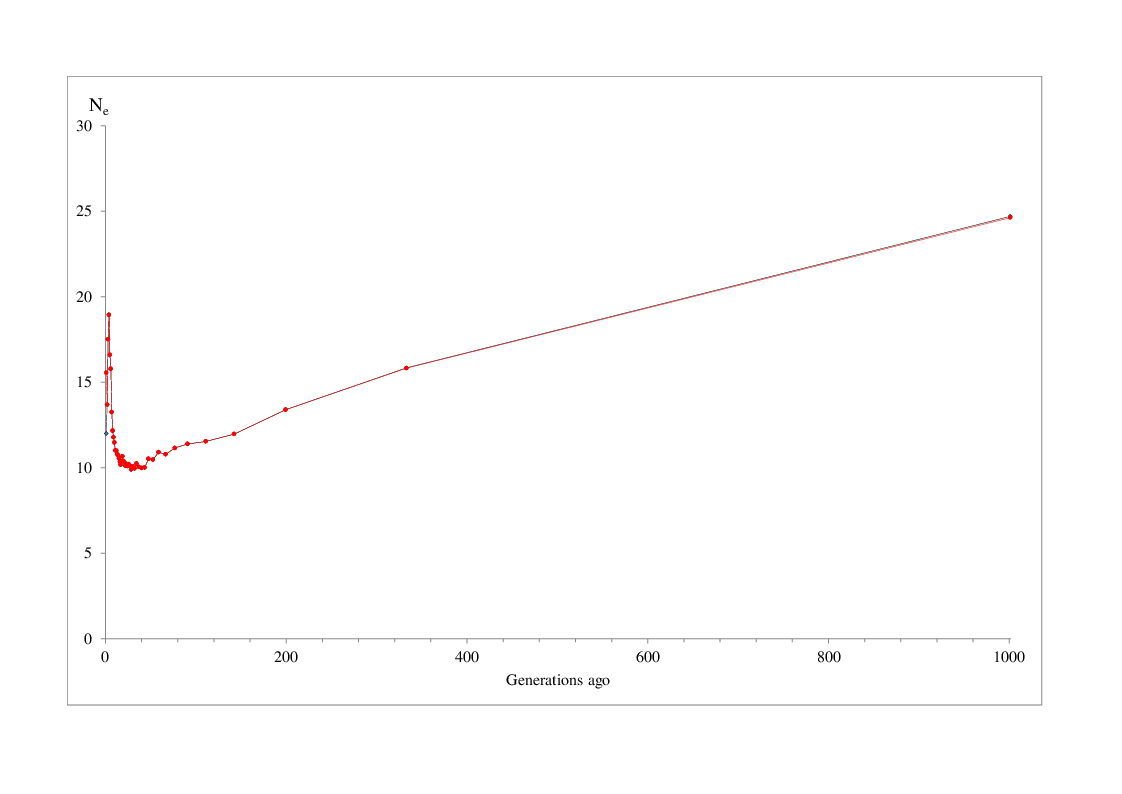

Supplement: S5 Fig — The effective population size (Ne) was estimated from the mean r2 for the 38 canine autosomes and after filtering for a minor allele frequency (MAF) <0.01 in a multibreed dog panel. The black graph shows Ne without restriction due to MAF and the red graph represents Ne after restriction due to a MAF<0.01. (TIF) [file pone.0122680.s005.tif]

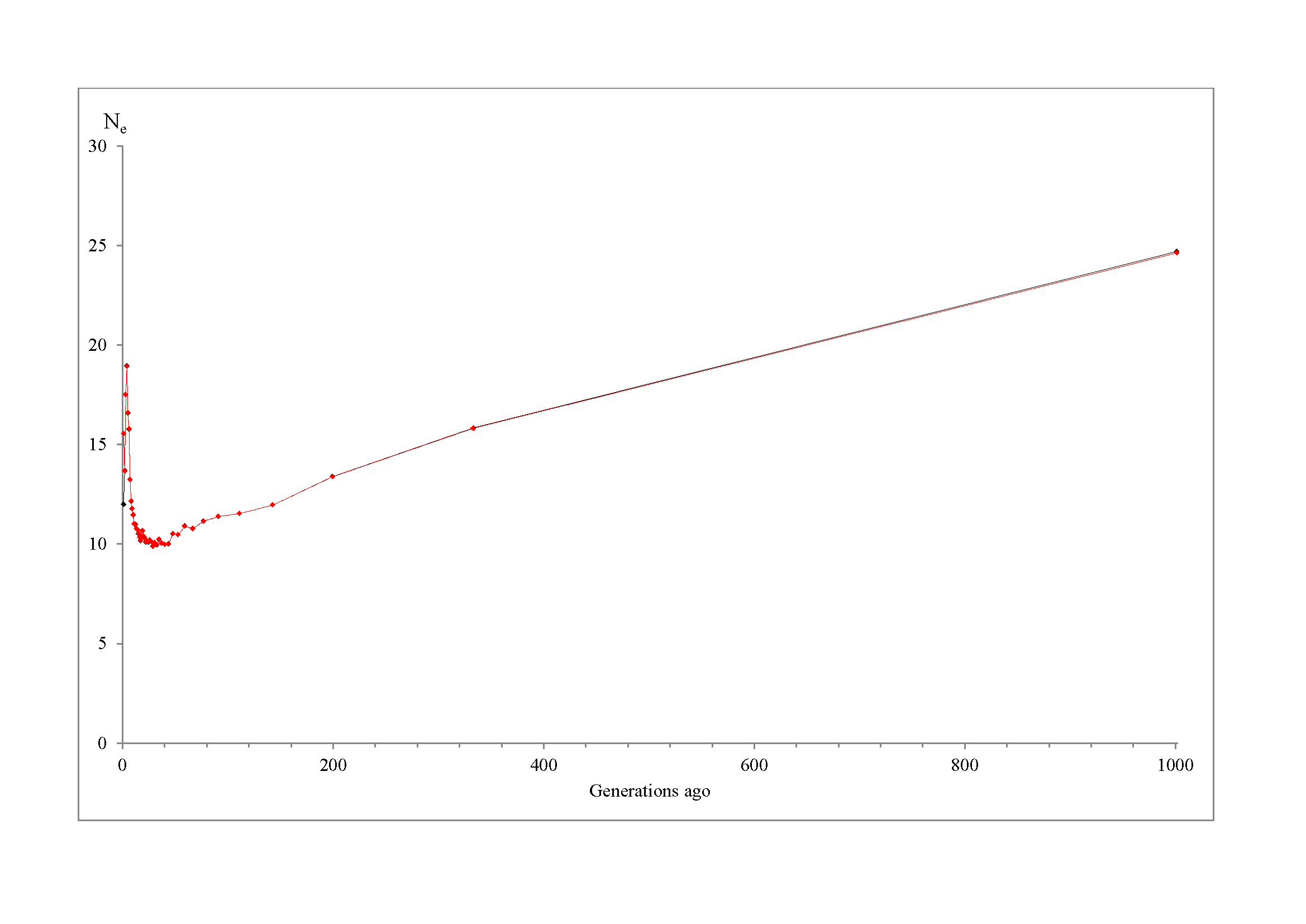

Supplement: S6 Fig — The effective population size (Ne) was estimated from the mean r2 for the 38 canine autosomes and after filtering for a minor allele frequency (MAF) <0.05 in a multibreed dog panel. The black graph shows Ne without restriction due to MAF and the red graph represents Ne after restriction due to a MAF<0.05. (TIF) [file pone.0122680.s006.tif]
