# Supplementary material for: Effective Population Size, Extended Linkage Disequilibrium and Signatures of Selection in the Rare Dog Breed Lundehund
Source: PLoS One. 2015 Apr 10;10(4):e0122680. doi: 10.1371/journal.pone.0122680 (PMC4393028; doi:10.1371/journal.pone.0122680)
Supplement: S2 Table — Given are the minimum length of ROHs, their equivalence in number of generations back (GEN), the inbreeding coefficients FROH_50SNP, FROH_65SNP and FROH_358SNP with their standard deviations (SD) and Pearson correlation coefficients with the respective FROH without restrictions for minimum length. (DOCX) [file pone.0122680.s008.docx]

**S2 Table. Runs of homozygosity (ROHs) for 50-, 60- and 358-SNP-thresholds and with different restrictions for minimum lengths of ROHs in Mb (MIN-ROH).** Given are the minimum length of ROHs, their equivalence in number of generations back (GEN), the inbreeding coefficients F_ROH_50SNP,_ F_ROH_65SNP and_ F_ROH_358SNP_ with their standard deviations (SD) and Pearson correlation coefficients with the respective F_ROH_ without restrictions for minimum length.

| MIN-ROH (Mb) | GEN | F_ROH_50SNP_ | r-F_ROH_50SNP_ | F_ROH_65SNP_ | r-F_ROH_65SNP_ | F_ROH_358SNP_ | r-F_ROH_358SNP_ |
| --- | --- | --- | --- | --- | --- | --- | --- |
|  |  | Mean ± SD |  | Mean ± SD |  | Mean ± SD |  |
| >1.0 | 50 | 0.87±0.017 | 1.0 | 0.87±0.018 | 1.0 | 0.81±0.066 | 1.0 |
| >2.0 | 25 | 0.86±0.028 | 0.90 | 0.86±0.028 | 0.90 | 0.81±0.066 | 1.0 |
| >2.5 | 20 | 0.85±0.035 | 0.84 | 0.85±0.035 | 0.84 | 0.81±0.066 | 1.0 |
| >3.3 | 15 | 0.84±0.046 | 0.78 | 0.84±0.046 | 0.78 | 0.81±0.066 | 1.0 |
| >5.0 | 10 | 0.80±0.068 | 0.68 | 0.80±0.068 | 0.68 | 0.80±0.069 | 1.0 |
| >5.5 | 9 | 0.79±0.074 | 0.65 | 0.79±0.074 | 0.65 | 0.79±0.074 | 1.0 |
| >6.25 | 8 | 0.77±0.084 | 0.66 | 0.77±0.084 | 0.67 | 0.77±0.084 | 1.0 |
| >7.14 | 7 | 0.75±0.093 | 0.65 | 0.75±0.093 | 0.66 | 0.75±0.093 | 0.99 |
| >8.33 | 6 | 0.73±0.111 | 0.63 | 0.73±0.111 | 0.64 | 0.73±0.111 | 0.99 |
| >10.0 | 5 | 0.70±0.126 | 0.64 | 0.70±0.126 | 0.65 | 0.70±0.126 | 0.98 |
| >12.5 | 4 | 0.64±0.143 | 0.60 | 0.64±0.143 | 0.60 | 0.64±0.143 | 0.96 |
| >16.7 | 3 | 0.53±0.151 | 0.57 | 0.53±0.151 | 0.58 | 0.53±0.151 | 0.93 |
| >25.0 | 2 | 0.37±0.135 | 0.53 | 0.37±0.134 | 0.54 | 0.37±0.135 | 0.86 |
| >50.0 | 1 | 0.10±0.042 | 0.43 | 0.10±0.042 | 0.43 | 0.10±0.042 | 0.60 |
