# Supplementary material for: Effective Population Size, Extended Linkage Disequilibrium and Signatures of Selection in the Rare Dog Breed Lundehund
Source: PLoS One. 2015 Apr 10;10(4):e0122680. doi: 10.1371/journal.pone.0122680 (PMC4393028; doi:10.1371/journal.pone.0122680)
Supplement: S3 Table — The mean minor allele frequency (MAF) over all 157,423 autosomal SNPs in the Lundehund, the proportion and mean number of informative SNPs (MAF >0.0), the fixation index (FIS) and the mean number of observed and expected homozygous SNPs are given. (DOCX) [file pone.0122680.s009.docx]

**S3 Table. Genetic diversity of Lundehund genotyped using the Illumina Canine High Density Beadchip.** The mean minor allele frequency (MAF) over all 157,423 autosomal SNPs in the Lundehund, the proportion and mean number of informative SNPs (MAF >0.0), the fixation index (F_IS_) and the mean number of observed and expected homozygous SNPs are given.

| Parameter | Value |
| --- | --- |
| MAF | 0.026 |
| Proportion of informative SNPs | 0.111 |
| Mean number of informative SNPs | 17,410 |
| F_IS_ | -0.071 |
| Mean number of observed homozygous SNPs | 11,500.50 |
| Mean number of expected homozygous SNPs | 11,885.36 |
